# ABA and Ethylene Mediates Tomato Root Development Modulation During Endophytic Fungal Interaction

**DOI:** 10.3390/jof11100707

**Published:** 2025-09-30

**Authors:** Maria Feka, Bilge Chousein, Olga Tsiouri, Kalliope K. Papadopoulou

**Affiliations:** Plant and Environmental Biotechnology Laboratory, Department of Biochemistry and Biotechnology, University of Thessaly, Biopolis, 41500 Larissa, Greece; feka@uth.gr (M.F.); bchousein@uth.gr (B.C.); otsiouri@uth.gr (O.T.)

**Keywords:** *Fusarium solani* FsK, tomato, ethylene, ABA, endophyte, root development, hormone signaling

## Abstract

The early stages of plant–microbe interaction are critical for establishing beneficial symbioses. We investigated how the endophytic fungus *Fusarium solani* strain FsK modulates tomato (*Solanum lycopersicum*) development and hormone pathways during in vitro co-cultivation. Seedlings were sampled at three early interaction stages (pre-contact, T1; initial contact, T2, 3 days post-contact, T3). Root traits and root and leaf transcripts for abscisic acid (ABA) and ethylene (ET) pathways were quantified, alongside fungal ET-biosynthesis genes. FsK altered root system architecture, increasing root area, lateral root number, root-hair length, and fresh biomass. These morphological changes coincided with tissue- and time-specific shifts. In leaves, FsK broadly affected ABA biosynthetic and homeostasis genes (*ZEP1*, *NCED1*, *ABA2*, *AAO1*, *ABA-GT*, *BG1*), indicating reduced de novo synthesis with enhanced deconjugation of stored ABA. ET biosynthesis was curtailed in leaves via down-regulation of ACC oxidase (*ACO1–3*), with isoform-specific changes in ACC synthase (*ACS*). The ET receptor ETR1 was transiently expressed early (T1–T2). FsK itself showed staged activation of fungal ET-biosynthesis genes. These results reveal coordinated fungal–plant hormone control at the transcriptional level that promotes root development during early interaction and support FsK’s potential as a biostimulant.

## 1. Introduction

Given the massive difficulties that continue to limit the yield of agriculture, increasing crop production in this day and time requires improving productivity while maintaining sustainability and avoiding harm to the ecological system [1]. The use of microbial inoculants to promote plant development and mitigate biotic and abiotic stressors in planted crops is a less expensive, more environmentally friendly, and more sustainable way to achieve agricultural intensification and increase production [2].

In the research field of plant-microbe interactions, endophytic fungi are gaining interest. These fungi live inside plant tissues and appear to be at no harm to their host [3]. With their host plants, they can establish a symbiotic or mutual relationship. Endophytic microorganisms have been associated with many of the advantageous characteristics and functions of their plant host, such as boosting plant growth, suppressing plant pathogens, increasing plant tolerance to stress, and boosting plant immunity [1,3,4,5].

The ability of several kinds of endophytes to create growth-promoting compounds that are comparable to those produced by their host plants but in greater amounts has been investigated [6]. By protecting their plant host from environmental stimuli and controlling the synthesis of crucial phytohormones, endophytes also increase productivity by affecting the host plant’s physiological response. It has also been shown that fungal endophytes are significant providers of chemical compounds with metabolic activity [7,8]. Important plant hormones that are necessary for promoting plant development, like gibberellic acid, ethylene, and indole-3-acetic acid, can be produced by these microbes [8,9]. Among other factors, they help plants cope with drought stress and combat pathogens that cause plant diseases [10,11].

Endophytic fungi have been found to affect plant levels of abscisic acid (ABA), a phytohormone that is essential for stress responses, in addition to their growth-promoting abilities. Khan et al. (2010) showed a significantly lower ABA level in endophyte-colonized plants over their endophyte-free counterparts [12]. Similar findings were reported by Jahromi et al. (2008) in lettuce plants linked to *Glomus intraradices* [13]. Richardson et al. (2009) and Khan et al. (2011) showed lower ABA concentrations when endophytic fungi that produce phytohormones, such as *Aspergillus fumigatus* and *Penicillium funiculosum*, were present [14,15]. Along these lines, several other cases of microbes suppressing phytohormone levels have been reported [16,17]. On the other hand, through the phytohormones ethylene (ET) and jasmonic acid (JA), endophytes activate Induced Systemic Resistance (ISR), which leads to a more robust and rapid immune response after a pathogen attack. Some endophytes can stimulate host defenses and cause a salicylic acid (SA)-dependent Systemic Acquired Resistance response (SAR) [18,19,20].

ABA orchestrates abiotic responses in plants: beyond its hallmark role in closing stomata, it broadly modulates plant–water relations. Elevated ABA typically suppresses shoot growth while promoting primary root elongation and inhibiting lateral root formation to enhance water foraging—though these growth effects can vary with ABA concentration and species [21]. In plastids, zeaxanthin epoxidase (ZEP) converts zeaxanthin to violaxanthin, which is isomerized to 9-cis-epoxycarotenoids. The rate-controlling cleavage is catalyzed by 9-cis-epoxycarotenoid dioxygenases (NCEDs), generating xanthoxin that is exported to the cytosol. There, ABA2 (a short-chain dehydrogenase/reductase) oxidizes xanthoxin to abscisic aldehyde, and abscisic aldehyde oxidase (AAO; *AAO1* in tomato), with the Mo-cofactor supplied by ABA3, completes the final step to ABA [21,22]. ABA levels are then dynamically tuned by (i) catabolism via *CYP707A* (ABA 8′-hydroxylase) to phaseic acid, (ii) conjugation by UDP-glucosyltransferases (*ABA-GT/UGT*) to the storage form ABA-glucose ester (ABA-GE), and (iii) rapid remobilization of active ABA through β-glucosidases (*BG1/BG2*) that hydrolyze ABA-GE, largely from endomembrane/vacuolar pools [21,22].

Ethylene biosynthesis is initiated by precursors S-adenosyl-L-methionine (SAM) and 1-aminocyclopropane-1-carboxylic acid (ACC), and the key synthesizing enzymes catalyzing this route include SAM, ACC synthase (ACS), and ACC oxidase (ACO) [23]. The way that ethylene is produced in response to various limitations on the environment suggests a link between developmental adaptability and environmental change. Furthermore, ethylene functions as a signaling molecule that affects the molecular connections between plant development and other phytohormones. Examples of abiotic factors that cause ethylene production are drought, floods, submersion, shade, heat, exposure to heavy metals, salt, and a decrease in nutrition availability [24,25,26,27].

*Fusarium solani* strain FsK is a root-colonizing endophytic fungus with documented influence on host hormone dynamics [28]. Previous studies have shown that FsK increases the tolerance of tomato to drought stress [29] and exhibits strong phytoprotective capacity against biotic agents, including pathogens and herbivorous pests; transcriptional control of key genes involved in hormone-mediated responses was also reported [30,31]. The strain’s genome annotation has revealed genes encoding *SAM synthase*, *ACC synthase*, and *ACC oxidase*, suggesting the presence of a potential pathway for ethylene biosynthesis in the fungus. This predicted capacity, together with FsK’s ability to modulate host ABA and ET signaling, positions it as a promising model for studying interkingdom hormonal cross-talk.

While many studies have examined either plant or microbial gene expression during colonization, few have simultaneously profiled both partners’ hormone-related transcriptional changes in a temporally resolved manner. While auxin and cytokinin are primary regulators of root morphogenesis, we prioritized ABA and ethylene (ET) given evidence that FsK enhances drought-stress tolerance and because early microbe-induced and stress-primed host responses are mediated through ABA/ET, which directly shape root system architecture [21,23,24,25,26,27]. In this in vitro study, we investigate the early-stage interaction between FsK and tomato (*Solanum lycopersicum*) seedlings, integrating morphological analyses of root development with time-specific expression profiling of ABA- and ethylene-related genes in both tomatoes’ tissues and the fungus. This dual-focus approach provides new mechanistic insight into how an endophytic fungus coordinates its own hormone biosynthesis genes with host signaling to shape root architecture during colonization.

## 2. Materials and Methods

### 2.1. In Vitro Plant–Fungus Co-Cultivation and Sampling

Tomato seeds (*Solanum lycopersicum* cv. Moneymaker) were surface sterilized in 4% NaOCl for 10 min and rinsed six times with sterile deionized water. Seeds were soaked overnight at 4 °C to synchronize germination, then transferred to half-strength Murashige and Skoog (MS) medium [32] and incubated under controlled growth chamber conditions (16 h light/8 h dark, 22 °C).

The endophytic fungus *Fusarium solani* strain FsK [28] was cultured on potato dextrose agar (PDA) at 26 °C in darkness for 4 days [33]. Hyphal plugs (4 mm diameter) were placed ~1 cm away from the root tips of 7-day-old tomato seedlings. Control seedlings received no fungal inoculum. Plants were sampled at three time points: (T1) prior to contact, when roots were ~1 cm from the fungal colony; (T2) upon initial contact; and (T3) three days post-contact. Root systems were observed stereomicroscopically to document morphological changes.

### 2.2. Gene Selection

Genes were selected to represent critical enzymatic and regulatory steps in the abscisic acid (ABA) and ethylene (ET) pathways relevant to responses to *Fusarium solani* strain FsK. Isoform choice was based on the tomato (*Solanum lycopersicum*) genome annotations from the Sol Genomics Network and on isoform-specific functional studies in tomato stress physiology.

For the ethylene biosynthesis pathway, *ACS4* and *ACS8* were selected among the nine annotated ACC synthase (ACS) isoforms because they are well-documented stress-inducible members responsive to abiotic stress and ABA–ethylene crosstalk. *ACS4* is modulated by ABA-responsive transcription factors during stress and developmental processes [34,35], while *ACS8* fine-tunes ethylene levels under drought and osmotic stress [22]. For ACC oxidase (ACO), all three transcriptionally active isoforms (*ACO1*, *ACO2*, and *ACO3*) were included to capture the full spectrum of regulation at the terminal step of ethylene biosynthesis. *ACO1* is strongly induced in leaves under abiotic stress and by ABA [36]; *ACO2* is stress-responsive under osmotic stress [37]; and because *LeERF2/TERF2* directly binds the DRE/CRT element in the *ACO3* promoter and activates transcription, *ACO3* is positioned within a positive ethylene feedback loop relevant to stress signaling [38].

For the ABA pathway, the following genes were selected: *ZEP1*, *NCED1*, *ABA2*, and *AAO1* (biosynthesis) [39]; *CYP707A1* (catabolism) [35,40,41,42]; *ABA-GT/UGT75C1* (conjugation) [35,41,42,43,44]; and *BG1* (vacuolar release) [22,45,46]. These represent major control points in ABA homeostasis and have been implicated in systemic stress signaling.

Isoform-specific primers were designed using *S. lycopersicum* sequences from the Sol Genomics Network. Previously published primers were adopted where available—*LeUbi*, *NCED1*, *AAO1*, *UGT75C1*, *ACS4*, *ACS8*, *ACO2*, *ACO3*, *ETR1*, and *ACO1*—with sequences and references listed in Supplementary Table S1. Primers designed in this study (e.g., *BG1*, *CYP707A1*, and FsK-specific genes) are indicated as “This work” in Supplementary Table S1.

### 2.3. Gene Expression, Root Trait Measurement

Total RNA was extracted from leaves and roots using LiCl precipitation. Quantitative RT-PCR was performed on 10 ng RNA per reaction using the Luna Universal One-Step RT-qPCR Kit (New England Biolabs, Ipswich, MA, USA). The tomato ubiquitin (*LeUbi*) gene was used as a reference, and *Translation Elongation Factor 1-α* (*Tef1-α*) as the fungal housekeeping gene. Primer sequences are listed in Supplementary Table S1. Root area and root hair length were quantified using ImageJ v1.54 (bundled with 64-bit Java 8; NIH, Bethesda, MD, USA) based on calibrated digital images. Lateral root number and fresh weight were also recorded at each time point.

### 2.4. Statistical Analysis

All statistical analyses were performed in R (version 4.x, 2023). Data were first tested for normality (Shapiro–Wilk test) and homogeneity of variances (Levene’s test). When the assumption of homoscedasticity was not met, a Box–Cox transformation was applied using the caret package (caret 6.0-94). For multi-group comparisons, ANOVA followed by Tukey’s Honestly Significant Difference (HSD) post hoc test was applied. All differences were considered statistically significant at *p* < 0.05. Graphs were generated using the ggplot2 (2 3.4.3) and ggpubr (0.6.0) packages.

## 3. Results

### 3.1. FsK Alters Root Architecture During Early Interaction

**Figure 1:**
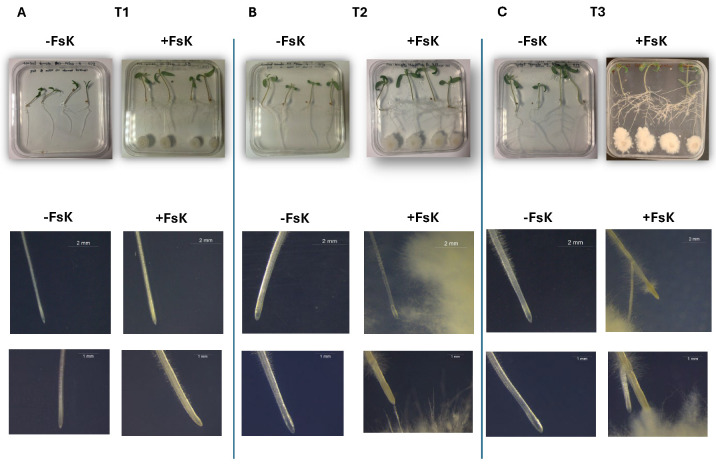
Early interaction of *Fusarium solani* strain FsK with tomato seedlings. Tomato (*Solanum lycopersicum* cv. Moneymaker) seedlings were grown on half-strength MS medium in vitro either without (-FsK) or with (+FsK) inoculation by *F. solani* FsK. Plants were imaged at three stages: T1, pre-contact (root tips ~1 cm from the fungal colony) (**A**); T2, initial contact (**B**); and T3, 3 days post-contact (**C**). The insets below each plate show the corresponding root tips (scale bars = 2 mm and 1 mm). FsK-treated seedlings exhibited enhanced root system development—greater primary root elongation, increased lateral root emergence, and dense root-hair proliferation—most evident at T2–T3. White mycelial growth at the plate base marks the fungal colonies in +FsK treatments. Images are representative of five independent plates per condition.

**Figure 2:**
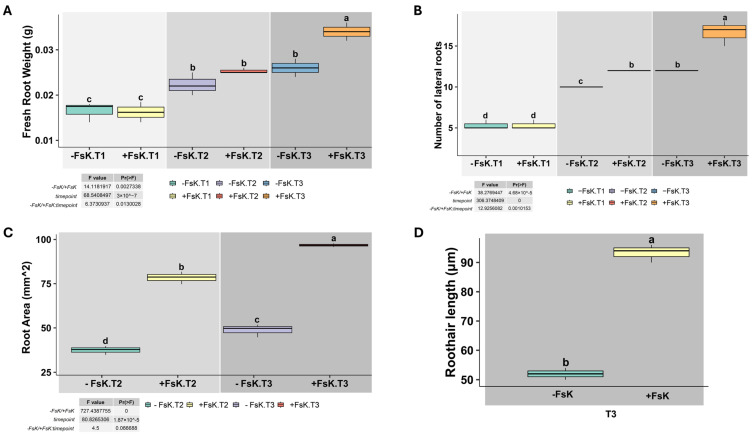
Effect of *Fusarium solani* strain FsK on tomato root growth parameters during early interaction stages. Boxplots show (**A**) fresh root weight, (**B**) number of lateral roots, (**C**) root area, and (**D**) root hair length in tomato (*Solanum lycopersicum* cv. Moneymaker) seedlings grown in vitro without (-FsK) or with (+FsK) inoculation of *Fusarium solani* strain FsK. Measurements were taken at three time points: T1, pre-contact; T2, initial contact; and T3, three days post-contact. Data represent means ± standard deviation (n = 3). Different letters above boxplots indicate statistically significant differences according to ANOVA followed by Tukey’s HSD test (*p* < 0.05). Insets display F and *p* values for the effects of treatment, time point, and their interaction.

Phenotypic and stereoscopic observations showed clear differences between FsK-treated and control plants, indicating that the endophyte exerts a sustained effect on root architecture (Figure 1). The root fresh weight, number of lateral roots, root area, and root hair length were measured to better understand the effect of the endophyte’s presence during the early phases of interaction (Figure 2). Root fresh weight did not vary among treatments at T1; however, by T3, the difference became pronounced and statistically significant, suggesting that the endophyte contributes to increased root biomass accumulation as the interaction progresses (Figure 2A). Lateral root number was significantly affected by FsK treatment. Co-inoculated plants showed a clear and statistically significant increase in lateral root numbers at both T2 and T3 compared to the control (Figure 2B). This trend suggests that, under our experimental conditions, FsK actively stimulates lateral root development, particularly during the later stages of the interaction.

Compared to the control, FsK-treated plants exhibited a noticeable increase in root area at T2, suggesting that FsK promotes early root development. Root area continued to increase at T3 in the presence of FsK (Figure 2C). At T3, FsK-inoculated plants exhibited significantly longer root hairs than the control group, indicating that the fungus promotes root hair elongation, potentially improving nutrient and water uptake (Figure 2D).

To further investigate the mechanisms by which FsK influences plant hormone homeostasis, gene expression analyses were performed at the early stages of the interaction for key enzymes involved in ethylene and ABA biosynthesis.

### 3.2. Tissue-Specific Modulation of ABA and Ethylene-Related Gene Expression in Tomato During Early Interaction with FsK

**Figure 3:**
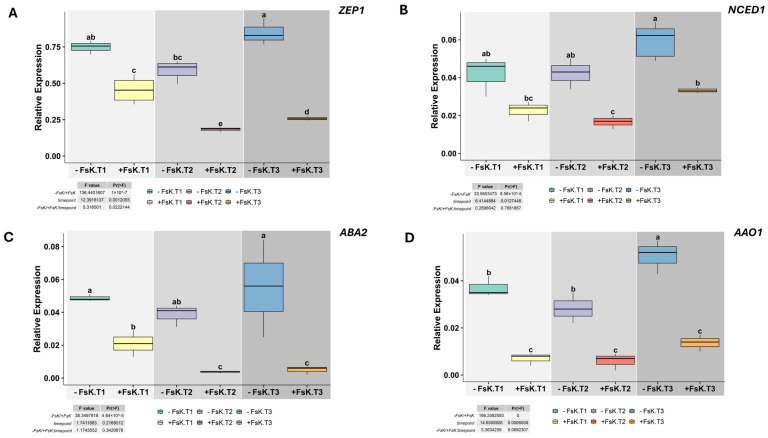
Expression of *ZEP1*, *NCED1*, *ABA2* and *AAO1* in tomato leaves during early interaction with *Fusarium solani* strain FsK. Relative transcript levels of *ZEP1* (**A**), *NCED1* (**B**), *ABA2* (**C**), and *AAO1* (**D**) in leaves of tomato (*Solanum lycopersicum* cv. Moneymaker) seedlings grown in vitro without (-FsK) or with (+FsK) inoculation. Measurements were taken at three early interaction stages: T1, pre-contact (roots ~1 cm from fungal colony); T2, initial hyphal contact; and T3, three days post-contact. Data represent means ± standard deviation (n = 3). Different letters above boxplots indicate statistically significant differences according to ANOVA followed by Tukey’s HSD test (*p* < 0.05). Insets display F and *p* values for the effects of treatment, time point, and their interaction.

In the present in vitro study, across all time points, *ZEP1* and *NCED1* transcript levels were consistently lower in *FsK*-treated leaves than in controls (Figure 3A,B). In FsK-treated plants’ roots, both genes showed statistically significant elevation at T3, suggesting that FsK enhances ABA biosynthesis in the root (Supplementary Materials Figure S1A,B). In control plants, *ABA2* and *AAO1* remained high throughout, whereas FsK-treated leaves exhibited reduced expression of both genes, consistent with an endophyte-mediated dampening of ABA biosynthesis (Figure 3C,D). *ABA2* expression in FsK-treated roots remained modest and stable, indicating a potential dampening effect by the endophyte (Supplementary Materials Figure S1C). At T2 and T3, FsK also induced a downregulation of *AAO1*, a gene involved in the final step of ABA biosynthesis. This suggests that FsK actively inhibits *AAO1*, possibly as a mechanism to alter plant hormone signaling.

A systemic effect was evident: across all time points, FsK-treated plants displayed suppressed expression of ABA-related genes in leaves. This indicates that the FsK–tomato interaction induces a systemic response affecting ABA metabolism and signaling at the whole-plant level.

**Figure 4:**
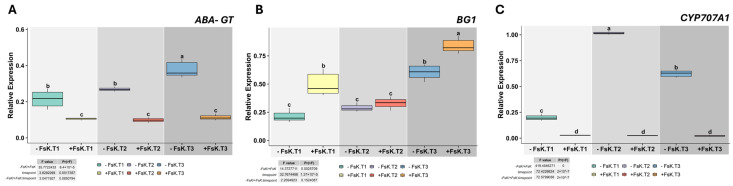
Expression of *ABA-GT*, *BG1* and *CYP707A1* in tomato leaves during early interaction with *Fusarium solani* strain FsK. Relative transcript levels of *ABA-GT* (**A**), *BG1* (**B**), and *CYP707A1* (**C**) in leaves of tomato (*Solanum lycopersicum* cv. Moneymaker) seedlings grown in vitro without (-FsK) or with (+FsK) inoculation. Measurements were taken at three early interaction stages: T1, pre-contact (roots ~1 cm from fungal colony); T2, initial hyphal contact; and T3, three days post-contact. Data represent means ± standard deviation (n = 3). Different letters above boxplots indicate statistically significant differences according to ANOVA followed by Tukey’s HSD test (*p* < 0.05). Insets display F and *p* values for the effects of treatment, time point, and their interaction.

*ABA-GT*, which encodes an ABA-glucosyltransferase responsible for converting ABA into its inactive storage form (ABA-glucose ester), was downregulated in both roots and leaves of FsK-inoculated plants, regardless of time point (Figure 4A, Supplementary Materials Figure S2A). In contrast, *BG1*, encoding β-glucosidase 1 that releases active ABA from storage in ER, was overexpressed in both tissues at T3 under FsK treatment (Figure 4B, Supplementary Materials Figure S2B). In FsK-treated plants, *CYP707A1*—which encodes ABA 8’-hydroxylase that breaks down ABA—expression showed no significant variation across treatments or time points in roots (Supplementary Materials Figure S2C) and was nearly undetectable in leaves (Figure 4C). These indicate that the endophyte does not directly influence *CYP707A1*-related ABA degradation. These findings suggest that FsK influences ABA homeostasis by manipulating biosynthesis while enhancing the reactivation of stored ABA.

**Figure 5:**
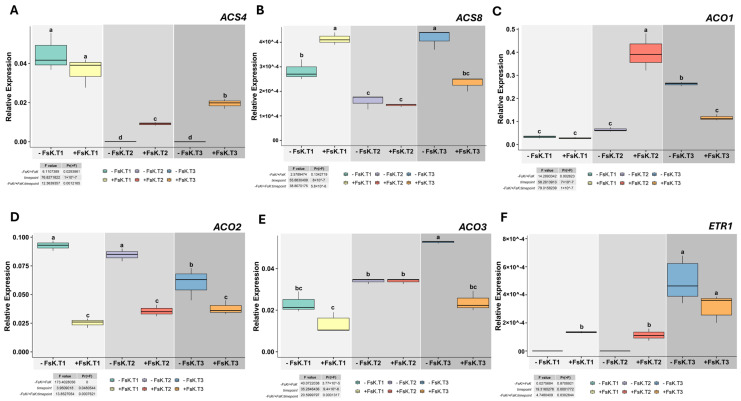
Expression of *ACS4*, *ACS8, ACO1*, *ACO2*, *ACO3,* and *ETR1* in tomato leaves during early interaction with *Fusarium solani* strain FsK. Relative transcript levels of *ACS4* (**A**), *ACS8* (**B**), *ACO1* (**C**)*, ACO2* (**D**)*, ACO3* (**E**), and *ETR1* (**F**) in leaves of tomato (*Solanum lycopersicum* cv. Moneymaker) seedlings grown in vitro without (-FsK) or with (+FsK) inoculation. Measurements were taken at three early interaction stages: T1, pre-contact (roots ~1 cm from fungal colony); T2, initial hyphal contact; and T3, three days post-contact. Data represent means ± standard deviation (n = 3). Different letters above boxplots indicate statistically significant differences according to ANOVA followed by Tukey’s HSD test (*p* < 0.05). The insets display F and *p* values for the effects of treatment, time point, and their interaction.

Analysis of ethylene biosynthesis and signaling genes further revealed tissue-specific responses to FsK. Expression patterns of *ACS4* and *ACS8* also differed between tissues. *ACS4* was overexpressed in roots and leaves of FsK-treated plants at T3, while *ACS8* expression was downregulated (Figure 5A,B, Supplementary Materials Figure S3A,B), suggesting their involvement in developmental or stress-related responses and pointing to a spatially distinct regulatory mechanism. Under the presence of the endophyte, in tomato roots, *ACO1* was overexpressed at all time points, and *ACO2* and *ACO3* were upregulated only at T1 (Supplementary Materials Figure S3C–E). In contrast, all three *ACO* genes were downregulated in leaves under FsK treatment, indicating a consistent suppression of ethylene biosynthesis in aerial tissues (Figure 5C–E).

The expression level of the key gene *ETR1 (encodes an endoplasmic-reticulum ethylene receptor)*, a core component of ethylene signaling, is shown in Figure 5F. In roots, *ETR1* expression tended to be higher when the FsK was present at T1, indicating a transient activation of ethylene perception during the early interaction phase (Supplementary Materials Figure S3F). *ETR1* was only expressed in leaves at T1 and T2, suggesting a shift toward the activation of ethylene signaling pathways during early stages of interaction (Figure 5F). This short-term expression pattern suggests that ethylene signaling is selectively triggered to meet the immediate demands of early contact, without imposing prolonged metabolic costs.

### 3.3. Temporal Regulation of Fungal Ethylene Biosynthesis Gene Expression During Host Interaction

**Figure 6:**
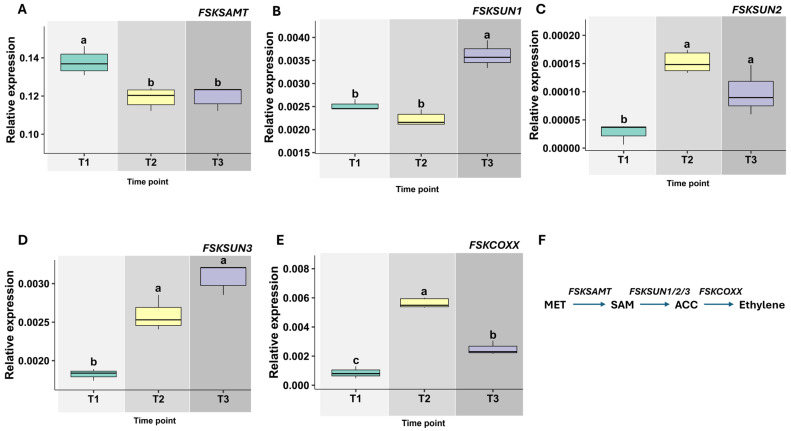
Expression of *Fusarium solani* strain FsK ethylene biosynthesis genes during early interaction with tomato. Relative transcript levels of *FSKSAMT* (**A**), *FSKSUN1* (**B**), *FSKSUN2* (**C**), *FSKSUN3* (**D**), and *FSKCOXX* (**E**) in FsK mycelium during in vitro co-cultivation with tomato (*Solanum lycopersicum* cv. Moneymaker) seedlings. Measurements were taken at three early interaction stages: T1, pre-contact (roots ~1 cm from fungal colony); T2, initial hyphal contact; and T3, three days post-contact. Data represent means ± standard deviation (n = 3). Data represent means ± standard deviation (n = 3). Different letters above boxplots indicate statistically significant differences according to ANOVA followed by Tukey’s HSD test (*p* < 0.05). (**F**) Proposed ethylene biosynthesis pathway in FsK, showing enzymatic steps catalyzed by *SAM synthase* (*FSKSAMT*), *ACC synthase* (*FSKSUN1–3*), and *ACC oxidase* (*FSKCOXX*).

Clear temporal regulation was observed in the expression of FsK genes involved in ethylene biosynthesis during its interaction with tomato roots. At T1, *FSKSAMT* (SAM synthase) was notably upregulated at the very beginning of the interaction. This persistent expression suggests an early activation and sustained role in the synthesis of ethylene or related metabolic precursors (Figure 6A).

In contrast, *FSKSUN1* (ACC synthase) showed increased expression later in the interaction, implying involvement in downstream processes or final-stage modulation of ethylene synthesis (Figure 6B). *FSKSUN2*, also encoding ACC synthase, exhibited its highest transcript levels at the mid-interaction stage (T2—at the initial contact), indicating a key role in maintaining ethylene production or signaling during this critical phase (Figure 6C). Similarly, *FSKSUN3* was upregulated at later time points, suggesting a function in the terminal stages of the interaction or downstream ethylene biosynthetic events (Figure 6D).

The expression of *FSKCOXX* (ACC oxidase), the gene responsible for the final step in ethylene biosynthesis, fluctuated throughout the time course. This dynamic expression pattern implies a regulatory role, potentially fine-tuning ethylene production in response to changing interaction demands (Figure 6E).

## 4. Discussion

Significant changes in root architecture and hormone signaling were observed during the interaction between tomato seedlings and the endophytic fungus *Fusarium solani* strain FsK. These findings provide new insight into the ways in which beneficial fungi influence plant development and hormone homeostasis. The observed changes reflect an intricate, temporally coordinated interaction between FsK and its host, comparable to mechanisms described in other well-characterized plant–fungal symbioses, including arbuscular mycorrhizal fungi, which synchronize gene expression with host root cell differentiation [47].

FsK significantly enhanced root area, lateral root number, root hair length, and root biomass across the time course of the experiment. These phenotypic changes suggest an active modulation of plant hormone pathways, particularly ethylene and abscisic acid (ABA). These hormone-driven morphological responses exemplify root developmental plasticity, a common adaptive mechanism enabling plants to optimize resource uptake under changing environmental and microbial conditions [48].

Comparable root architectural responses have been reported in plants interacting with other beneficial fungi. For instance, *Piriformospora indica* promotes lateral root proliferation in barley by influencing cytokinin and auxin pathways [49], while *Rhizophagus irregularis* enhances lateral root branching in *Medicago truncatula* by modulating auxin signaling [50]. In our in vitro study, FsK-treated plants exhibited pronounced root area expansion at T2 and T3, indicating a sustained growth-promoting effect. This aligns with findings on *Trichoderma virens*, which induces root elongation and branching in *Arabidopsis* via ethylene signaling [51]. The increased root hair length observed at T3 further supports a role for FsK in enhancing nutrient and water uptake, a trait also seen in maize roots colonized by *Rhizophagus intraradices* [52].

The gene expression data point to a complex modulation of ABA metabolism by FsK. Notably, FsK suppressed key biosynthesis genes such as *NCED1* and *AAO1* while upregulating *BG1*, which releases active ABA from storage forms. This mirrors findings from *Piriformospora indica*, which fine-tunes ABA homeostasis to enhance plant stress tolerance and root growth [53,54,55]. Modulation of ABA levels is a widespread strategy among endophytic fungi to mitigate stress-induced growth arrest and promote host resilience, particularly under drought or osmotic stress [56,57]. The strong upregulation of *BG1* in both roots and leaves suggests that FsK maintains active ABA pools through remobilization rather than synthesis.

Ethylene biosynthesis *ACO* and *ACS* family genes were also differentially regulated in FsK-treated plants. Ethylene has been widely implicated in regulating root elongation and branching during microbial symbioses [58,59,60]. Our observation that the ethylene signaling component (*ETR1*) was induced in leaves during the initial contact (T2) supports a tissue-specific regulatory strategy that may optimize local signaling while minimizing whole-plant stress responses. This localized hormonal regulation likely reflects an energy-efficient signaling strategy, as systemic activation of defense pathways is metabolically costly and often growth-inhibiting [61,62]. A similar pattern has been reported in the interaction between tomato and *Serendipita vermifera*, where ethylene signaling is enhanced locally in roots but restricted in shoots [63,64].

Collectively, the results indicate a clear division between local (root) and systemic (leaf) responses. In roots (local interface), FsK promoted the entry steps of ABA biosynthesis (*ZEP1*, *NCED1* ↑ at T3) and engaged the ethylene pathway (*ACO1* ↑ across time; *ACO2/3* transient ↑ at T1; *ACS4* ↑ and *ACS8* ↓), consistent with a pro-morphogenic, exploratory responsive root architecture program. In leaves (systemic tissue), FsK sustained a dampening of de novo ABA synthesis (*ZEP1*, *NCED1*, *ABA2*, *AAO1* ↓) and curtailed terminal ET production (*ACO1–3* ↓), with only a short early window of ET perception (*ETR1* at T1–T2). Across tissues, *ABA-GT* was reduced and *BG1* was induced at T3, while *CYP707A1* transcripts were low/near-undetectable in leaves—together indicating reduced ABA catabolism and greater reliance on remobilization of stored ABA rather than new synthesis. We note that we did not quantify ABA/ET concentrations or metabolites; these inferences are based on expression of biosynthesis, catabolism/conjugation, deconjugation, and signaling genes and should be interpreted as correlative. We interpret this as a “local activation/systemic restraint” strategy: roots locally elevate ABA/ET programs to drive architectural remodeling, while leaves limit prolonged stress-hormone output to contain whole-plant costs. Our conclusions about ABA–ethylene homeostasis are based on transcript levels in roots, leaves, and root morphology, without direct quantification of endogenous hormones or their metabolites. We did not measure ethylene concentrations. Future work will quantify ABA and ethylene by LC–MS/MS (including conjugates/catabolites) and GC-based ethylene/ACC assays in both leaves and roots, link these measurements to tissue-resolved expression (e.g., root-specific qPCR/RNA-seq or reporter lines), and separate volatile from diffusible signaling using sealed/vented split-plate assays and conditioned media. These experiments will directly test the proposed model and define the spatial origin of the signals underlying the observed pre-contact response.

Finally, the temporal regulation of FsK’s own ethylene biosynthesis genes underscores its adaptive coordination during the interaction. Early induction of *FSKSAMT* likely serves as a priming mechanism, while mid- and late-stage peaks in *FSKSUN2*, *FSKSUN1*, and *FSKSUN3* support sustained and fine-tuned ethylene production. While ethylene production by fungi such as *Fusarium* and *Botrytis* has been previously reported [65,66], the stage-specific transcriptional regulation of ethylene biosynthesis genes during plant colonization remains poorly characterized, highlighting the novelty of this study. The fluctuating expression of *FSKCOXX* suggests a dynamic role in regulating ethylene flux in response to host cues. Together, these findings reveal a sophisticated and temporally controlled fungal strategy for modulating host responses through both endogenous signaling and fungal-derived hormone production.

## 5. Conclusions

Our in vitro findings demonstrate that the endophytic fungus *Fusarium solani* strain FsK significantly enhances tomato seedling development by modulating root architecture and hormonal signaling. FsK-treated plants exhibited increased root area, lateral root number, root hair length, and root biomass, highlighting its potential as a plant growth-promoting endophyte. Although our experimental setup only involved in vitro conditions, the observed modulation of ethylene and abscisic acid (ABA) biosynthesis and signaling pathways suggests that FsK employs a sophisticated strategy to optimize plant growth and physiological adaptability that warrants further investigation.

Key findings from this work align with established roles of other beneficial fungi, such as *Piriformospora indica*, *Trichoderma* spp., and *Rhizophagus irregularis*, which similarly promote root development and influence hormone dynamics. FsK also exhibits temporally regulated expression of its own ethylene biosynthesis genes, highlighting an example of direct microbial contribution to the hormonal signaling environment. This dynamic regulation underscores the mechanistic complexity of FsK’s interaction with its host. In addition, a systemic effect on leaf gene expression was observed even at the pre-contact stage (T1), suggesting that communication is mediated by diffusible or volatile signals. and future work will focus on this important parameter.

Finally, our findings support the potential of FsK as a biofertilizer or biostimulant for enhancing crop productivity and resilience under diverse environmental conditions. Its ability to promote root system architecture and modulate hormone signaling suggests potential applications in improving crop performance under stress conditions. By prioritizing early-stage growth promotion while maintaining long-term adaptive responses with minimal metabolic cost to the host, FsK exemplifies a beneficial endophyte with promising applications in sustainable agriculture. Future studies should evaluate its performance under field conditions and in combination with other plant-beneficial microbes.

## Data Availability

The original contributions presented in this study are included in the article/Supplementary Material. Further inquiries can be directed to the corresponding author.

## Supplementary Materials

The following supporting information can be downloaded at https://www.mdpi.com/article/10.3390/jof11100707/s1, Figure S1: Expression of *ZEP1*, *NCED1*, *ABA2* and *AAO1* in tomato roots during early interaction with *Fusarium solani* strain FsK. Figure S2: Expression of *ABA-GT*, *BG1* and *CYP707A1* in tomato roots during early interaction with *Fusarium solani* strain FsK. Figure S3: Expression of *ACS4*, *ACS8*, *ACO1*, *ACO2*, *ACO3* and *ETR1* in tomato roots during early interaction with *Fusarium solani* strain FsK. Table S1: Primers used in this study. Refs. [31,35,37,67,68,69] are cited in the Supplementary Materials.
