# Supplementary material for: ABA and Ethylene Mediates Tomato Root Development Modulation During Endophytic Fungal Interaction
Source: J Fungi (Basel). 2025 Sep 30;11(10):707. doi: 10.3390/jof11100707 (PMC12565129; doi:10.3390/jof11100707)
Supplement: Supplementary file 1 [file jof-11-00707-s001.zip › jof-3850774-supplementary.pdf]

Supplementary Table S1. Primers used in this study.

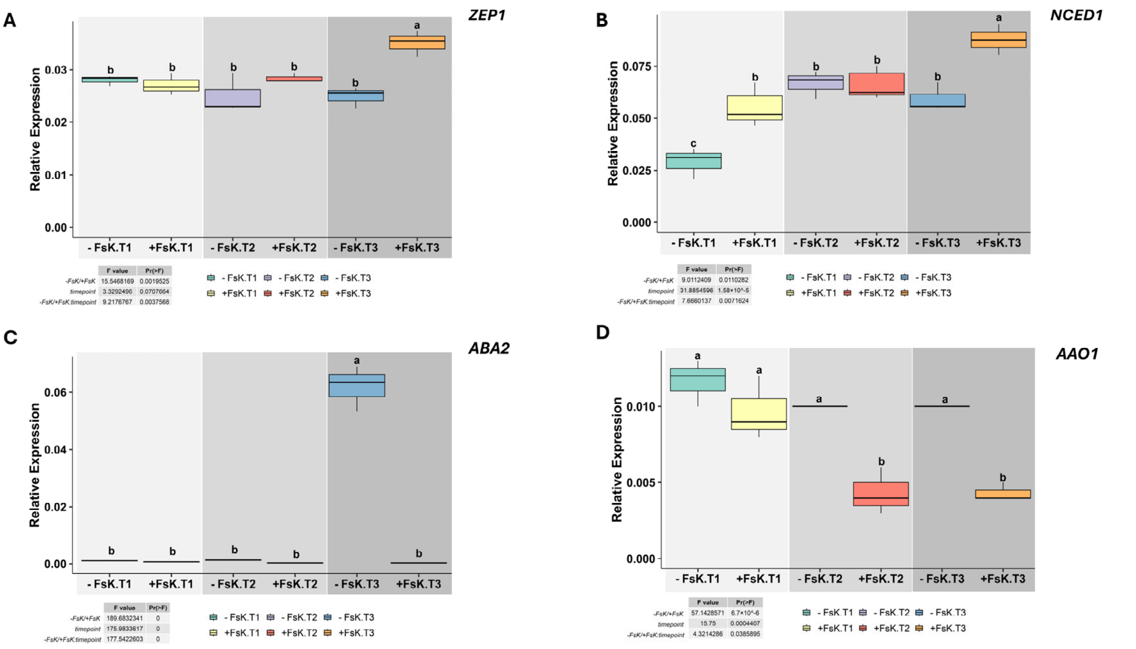

Figure S1. Expression of *ZEP1*, *NCED1, ABA2 and AAO1* in tomato roots during early interaction with *Fusarium solani* strain FsK. Relative transcript levels of *ZEP1* (A), *NCED1* (B), *ABA2* (C) and *AAO1* (D) in roots of tomato (*Solanum lycopersicum* cv. Moneymaker) seedlings grown *in vitro* without (−FsK) or with (+FsK) inoculation. Measurements were taken at three early interaction stages: T1, pre-contact (roots ~1 cm from fungal colony); T2, initial hyphal contact; and T3, three days post-contact. Data represent means ± standard deviation (n = 3). Different letters above boxplots indicate statistically significant differences according to ANOVA followed by Tukey's HSD test ($p < 0.05$). Insets display F and p values for the effects of treatment, timepoint, and their interaction.

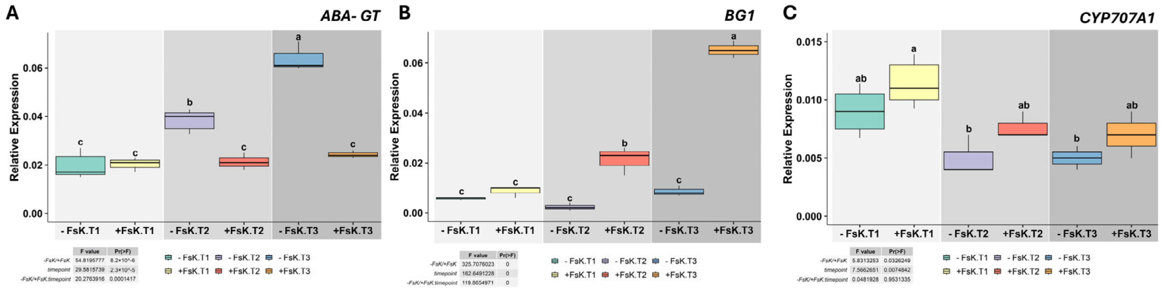

Figure S2. Expression of *ABA-GT*, *BG1* and *CYP707A1* in tomato roots during early interaction with *Fusarium solani* strain FsK. Relative transcript levels of *ABA-GT* (A) and *BG1* (B) and *CYP707A1* (C) in roots of tomato (*Solanum lycopersicum* cv. Moneymaker) seedlings grown *in vitro* without (−FsK) or with

(+FsK) inoculation. Measurements were taken at three early interaction stages: T1, pre-contact (roots ~1 cm from fungal colony); T2, initial hyphal contact; and T3, three days post-contact. Data represent means ± standard deviation (n = 3). Different letters above boxplots indicate statistically significant differences according to ANOVA followed by Tukey's HSD test ($p < 0.05$). Insets display F and p values for the effects of treatment, timepoint, and their interaction.

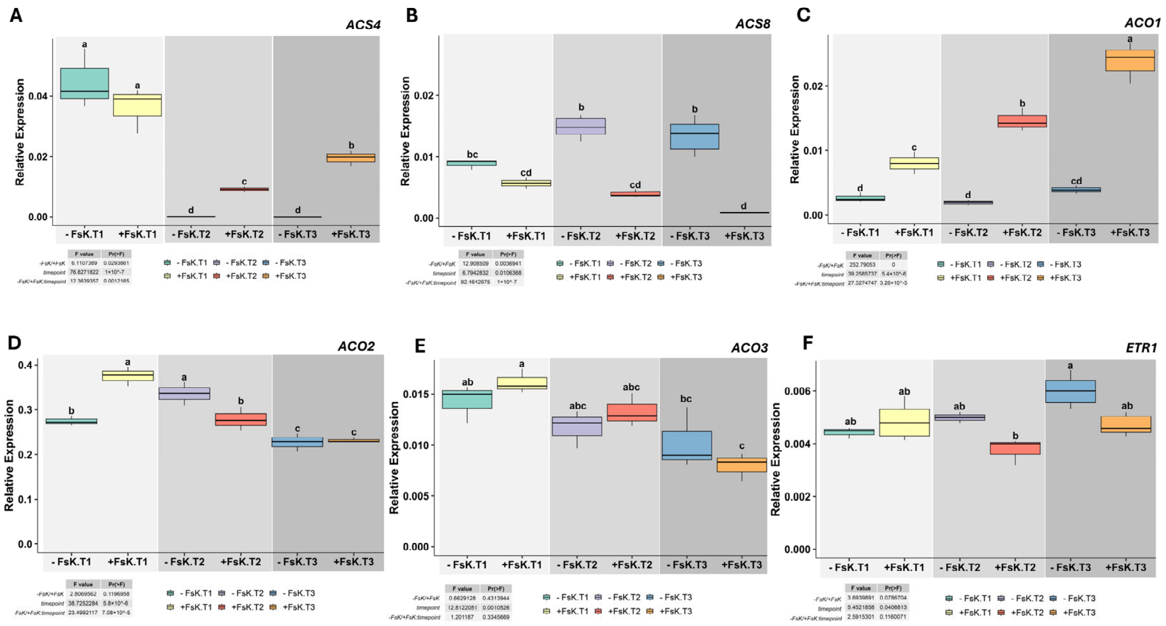

Figure S3. Expression of *ACS4*, *ACS8, ACO1*, *ACO2*, *ACO3* and *ETR1* in tomato roots during early interaction with *Fusarium solani* strain FsK. Relative transcript levels of *ACS4* (A), *ACS8* (B), *ACO1* (C)*, ACO2* (D)*, ACO3* (E) and *ETR1* (F) in roots of tomato (*Solanum lycopersicum* cv. Moneymaker) seedlings grown *in vitro* without (−FsK) or with (+FsK) inoculation. Measurements were taken at three early interaction stages: T1, pre-contact (roots ~1 cm from fungal colony); T2, initial hyphal contact; and T3, three days post-contact. Data represent means ± standard deviation (n = 3). Different letters above boxplots indicate statistically significant differences according to ANOVA followed by Tukey's HSD test ($p < 0.05$). Insets display F and p values for the effects of treatment, timepoint, and their interaction.
