# Supplementary material for: ABA and Ethylene Mediates Tomato Root Development Modulation During Endophytic Fungal Interaction
Source: J Fungi (Basel). 2025 Sep 30;11(10):707. doi: 10.3390/jof11100707 (PMC12565129; doi:10.3390/jof11100707)
Supplement: Supplementary file 1 [file jof-11-00707-s001.zip › TABLE S1.pdf]

Supplementary Table S1. Primers used in this study

Name	Sequence 5' to 3'	Reference
TEF1a	F: GGGTGGTTGAGGACAATGAC<br>R: CAGCTCACTGAGGGTCTTCC	This Work
Leubi	F: AAGATGGAAGAACTCTGGCG<br>R: TCACAACACATCACAAGGTC	[1]
ZEP1	F: GGTGGTGCGGATGCCCCAAA<br>R: GGCTGCATGGCATGGACGGA	[2]
NCED1	F: CTTATTTGGCTATCGCTGAACC<br>R: CCTCCAACTTCAAACTCATTGC	[3]
ABA2	F: GGTGGCCTAGGGCCTCATGC<br>R: TCCACGCCCTTGAGGTTCGC	[2]
AA01	F: GTGGAGGTTGGAGGAGGGGTTGATGTT<br>R: TGTGGTACTTCCAGCCGTTAATCCA	[3]
ABA-GT (UGT75C1)	F: CTTAAACTTGGCGGCATTCTCTGAT<br>R: ATATGGAGTTCACGCGCTACCTCAG	[4]
BG1	F: AATTACCGCGACGACTCTGC<br>R: GATTACATAGAGCCACAACATCTGC	This Work
CYP707A	F: AGAGAGGCTGTAGCTGAGTGG<br>R: TTGGCAAGTTCATTCCCTGGAC	This Work
ACS4	F: AAACGTGTAGTAATGGCTGGTG<br>R: AAATCCTGGGTAATAGGGTGTG	[5]
ACS8	F: AAACGTGTAGTAATGGCTGGTG<br>R: AAATCCTGGGTAATAGGGTGTG	[5]
ACO1	F: GGTTACTTGAAAAATGCCTTTTA<br>R: GTCTTTGAGGAGTTGAAGGCCA	[6]
ACO2	F: GTCATTAGCATCCTTCTACAATCCA<br>R: TGTTATGTTCCTCTGCCTCTTTATC	[5]
ACO3	F: TGATTACCAACGGGAAGTACAAG<br>R: CAATTAGAGATGGTGCTGGATAGA	[5]
ETR1	F: TTCAAGGATTAAAGGTTTTGGTGAT<br>R: ATCACATCCAAGGTGTGTAAGCA	[5]
FsKSAM	F: GACAACTTCGACCTCCGACCT<br>R: GACCGTTCTTAGCCGTCTGGA	This Work
FsKSUN1	F: CGTGCCTTCATCGGTGGTTC<br>R: GACCGTTCTTAGCCGTCTGGA	This Work
FsKSUN2	F: ACCTTTCTACCAGGGGCGAC<br>R: ACCGAGACTAACATATCCGCCA	This Work

FsKSUN3	F: ACTTCATCAGCGACGAGATTTATGC	This Work
	R: ACTCGAAAGGCACAGGAGGAG	
FsKCOXX	F: TTGATGCCGGAGATTACAATGGC	This Work
	R: CTTTTCCTTCACGCCTCCCG	
	R: AAATCCTGGGTAATAGGGTGTG	

References

1. Pappas, M.L.; Liapoura, M.; Papantoniou, D.; Avramidou, M.; Kavroulakis, N.; Weinhold, A.; Broufas, G.D.; Papadopoulou, K.K. The Beneficial Endophytic Fungus Fusarium Solani Strain K Alters Tomato Responses against Spider Mites to the Benefit of the Plant. *Front. Plant Sci.* **2018**, *9*, 1–17, doi:10.3389/fpls.2018.01603.
2. Du, M.; Zhai, Q.; Deng, L.; Li, S.; Li, H.; Yan, L.; Huang, Z.; Wang, B.; Jiang, H.; Huang, T.; et al. Closely Related NAC Transcription Factors of Tomato Differentially Regulate Stomatal Closure and Reopening during Pathogen Attack. *Plant Cell* **2014**, *26*, 3167–3184, doi:10.1105/tpc.114.128272.
3. Muñoz-Espinoza, V.A.; López-Climent, M.F.; Casaretto, J.A.; Gómez-Cadenas, A. Water Stress Responses of Tomato Mutants Impaired in Hormone Biosynthesis Reveal Abscisic Acid, Jasmonic Acid and Salicylic Acid Interactions. *Front. Plant Sci.* **2015**, *6*, 1–14, doi:10.3389/fpls.2015.00997.
4. Sun, Y.; Ji, K.; Liang, B.; Du, Y.; Jiang, L.; Wang, J.; Kai, W.; Zhang, Y.; Zhai, X.; Chen, P.; et al. Suppressing ABA Uridine Diphosphate Glucosyltransferase (SlUGT75C1) Alters Fruit Ripening and the Stress Response in Tomato. *Plant J.* **2017**, *91*, 574–589, doi:10.1111/tpj.13588.
5. Jegadeesan, S.; Beery, A.; Altahan, L.; Meir, S.; Pressman, E.; Firon, N. Ethylene Production and Signaling in Tomato (Solanum Lycopersicum) Pollen Grains Is Responsive to Heat Stress Conditions. *Plant Reprod.* **2018**, *31*, 367–383, doi:10.1007/s00497-018-0339-0.
6. Jia, H.; Chen, S.; Liu, D.; Liesche, J.; Shi, C.; Wang, J.; Ren, M.; Wang, X.; Yang, J.; Shi, W.; et al. Ethylene-Induced Hydrogen Sulfide Negatively Regulates Ethylene Biosynthesis by Persulfidation of ACO in Tomato under Osmotic Stress. *Front. Plant Sci.* **2018**, *871*, 1–11, doi:10.3389/fpls.2018.01517.
